# Long Noncoding RNA TUG1 Promotes the Function in ox-LDL-Treated HA-VSMCs via miR-141-3p/ROR2 Axis

**DOI:** 10.1155/2020/6758934

**Published:** 2020-05-29

**Authors:** Yu Tang, Jing Hu, Zhiying Zhong, Yanfeng Liu, Yunxia Wang

**Affiliations:** ^1^Department of Cardiology, People's Hospital of Jiangxi Province, 330002, Nanchang, China; ^2^Department of Cardiology, Fourth Affiliated Hospital of Nanchang University, 330002, Nanchang, China

## Abstract

**Background:**

Atherosclerosis (AS) is a common severe disease around the world. The merging paper reported that long noncoding RNAs (lncRNAs) took part in diversified pathological processes of AS, although the mechanism remains unknown. This study is aimed at uncovering the profile of lncRNA taurine-upregulated gene 1 (TUG1), which has biological function, and potential mechanism in AS progression *in vitro*.

**Methods:**

Oxidized low-density lipoprotein (ox-LDL) was used for AS model construction *in vitro*. Levels of lncRNA TUG1, miR-141-3p, and receptor tyrosine kinase-like orphan receptor 2 (ROR2) were detected by quantitative real-time polymerase chain reaction (qRT-PCR) in AS tissues or in ox-LDL-treated vascular smooth muscle cells (HA-VSMCs). The biofunctional effects were examined by 3-(4,5-dimethyl-2-thiazolyl)-2,5-diphenyl-2-H-tetrazolium bromide (MTT) and transwell assays. The expression of proliferation-related proteins (CyclinD1, Ki-67) and metastasis-associated proteins (*β*-catenin, Vimentin) and ROR2 in cells was determined by western blot analysis. The potential binding sites were predicted by starBase software online and confirmed by dual-luciferase reporter analysis.

**Results:**

The expression of TUG1 and ROR2 was promoted in AS tissues and ox-LDL-treated HA-VSMCs. While the low expression of miR-141-3p negatively correlated with that of TUG1 or ROR2 in AS tissues. Silencing of TUG1 inhibited the proliferation, migration, invasion, and metastasis in ox-LDL-treated HA-VSMCs. Moreover, the putative binding sites between miR-141-3p and TUG1 or ROR2 were predicted by starBase software online. Also, miR-141-3p deletion reversed the positive effects of TUG1 knockdown on cells. Besides, downregulation of miR-141-3p disrupted the biofunctional results from ROR2 silencing.

**Conclusion:**

TUG1 enhanced the progression of AS *in vitro* by regulating the miR-141-3p/ROR2 axis.

## 1. Introduction

Atherosclerosis (AS), a common chronic multifactorial vascular disease, is the primary class of cardiovascular disease (CVD) [1, 2]. CVD is known as a severe disease with a high rate of mortality and morbidity globally, accompanied with a variety of risk factors, such as endothelial damage, endothelial cell apoptosis, macrophage recruitment, accumulation of vascular smooth muscle cells (VSMCs), and proinflammatory cytokine generation [3]. The development of pathological proliferation and inflammatory response from VSMC may promote atherosclerosis and arterial restenosis [4]. Oxidized low-density lipoprotein (ox-LDL) is regarded to be an essential factor in the development of AS by facilitating endothelial dysfunction and accelerating the growth and migration of VSMCs [5].

Long noncoding RNAs (lncRNAs) are a category of long RNAs with a length of more than 200 nucleotides (nts), which have no translation capacity and affect gene expression during the transcriptional stage [6]. Emerging evidence suggested that lncRNAs acted as functional regulators in tumorigenesis [7], neurology [8], cardiovascular system [9], and the development of other diseases [10].

Recently, increasing evidence has suggested that targeting lncRNA taurine-upregulated gene 1 (lncRNA TUG1) could work as a new supplementary therapeutic strategy for AS [11]. Li et al. showed that TUG1 expression was increased in serum specimens from 38 patients with AS, compared with 24 healthy participants [12]. Also, the aberrant expression of TUG1 facilitated cell growth and inflammatory factor secretion and suppressed the apoptosis in ox-LDL-stimulated macrophages and VSMCs [11]. Mechanically, the increased proliferation and migration changes induced by the transfection of primary human umbilical vein endothelial cells (HUVECs) with TUG1 overexpression could be reversed by inhibiting the Wnt pathway [13]. However, little information has been investigated about the role of TUG1 and potential mechanism in AS progression.

LncRNA-miRNA-gene regulator networks have drawn great attention in vascular pathophysiology [14]. It is reported that miR-141 may play an important role in ox-LDL-induced abnormal proliferation of the VSMC. For instance, overexpression of PAPPA impaired the miR-141-induced inhibition of proliferation in the VSMCs [15]. Meanwhile, miRNA-141 was also found to activate the Wnt signaling pathway in esophageal cancer [16] and mesenchymal stem cells [17]. However, few studies have been reported in the cardiovascular field. The specific Wnt/receptor/coreceptor combinations are particularly important in dictating the resulting downstream signaling effects. ROR2 is critical for activation of the signaling pathway by Wnt5a. Wnt5a and ROR2 were significantly expressed in advanced atherosclerotic lesions and macrophages/foam cells within the plaque [18].

In this study, we explored the expression patterns of TUG1 in AS tissues or ox-LDL-treated HA-VSMCs and the biofunctional effects upon proliferation, migration, invasion, and metastasis in ox-LDL-treated HA-VSMCs. Moreover, the molecular mechanism of TUG1 involved in AS was further investigated in HA-VSMCs.

## 2. Materials and Methods

### 2.1. Clinical Samples

The experiment was authorized by the Ethics Committee of People's Hospital of Jiangxi Province and executed according to the Declaration of Helsinki principles. Tissue samples from AS patients (*n* = 30) and healthy volunteers (*n* = 30) were collected from People's Hospital of Jiangxi Province. All samples were preserved at -80°C for storage. Informed consents were provided by all participants.

### 2.2. Cell Culture, Administration, and Transfection

A human vascular smooth muscle cell (HA-VSMC) line was obtained from American Type Culture Collection (ATCC, Manassas, VA, USA), with 1% penicillin/streptomycin (Beyotime Biotechnology Company, Shanghai, China), cultured as previously described [19]. ox-LDL (Biosynthesis Biotechnology Company, Beijing, China) was used for AS model construction *in vitro*. To be specific, the cells were cultured in the medium with the presence of diverse dosages of ox-LDL (0 *μ*g/mL, 25 *μ*g/mL, 50 *μ*g/mL, and 75 *μ*g/mL) for 24 h and grown in the medium containing ox-LDL at a final concentration of 50 *μ*g/mL for 24 h [11]. Short hairpin RNA (shRNA) targeting TUG1 (sh-TUG1), shRNA targeting ROR2 (sh-ROR2), TUG1 overexpression plasmid (TUG1), miR-141-3p inhibitor (anti-miR-141-3p), miR-141-3p mimic (miR-141-3p), and controls (sh-NC, pcDNA, anti-miR-NC, and miR-NC) were obtained from GenePharma (Shanghai, China). A Lipofectamine 3000 (Invitrogen, Carlsbad, CA, USA) kit was used for transfection according to the manufacturer's instructions. The sequences were shown as follows: sh-TUG1, sequence, 5′-GACTACCTTCCCTGTGCTATT-3′; sh-ROR2, sequence, 5′-GCCCGATTCCAACTCTGAAAG-3′.

### 2.3. RNA Isolation and Quantitative Real-Time Polymerase Chain Reaction (qRT-PCR)

Total RNA from tissues and cells was extracted by using a TRIzol reagent (Thermo Fisher Scientific, Waltham, MA, USA) and reverse-transcribed using All-in-One™ miRNA PrimeScript™ RT reagent kit (Takara, Shiga, Japan) and PrimeScript RT reagent kit (Takara). qRT-PCR was performed on the 7500 Fast Real-Time PCR system (Thermo Fisher Scientific) with a qRT-PCR Detection Kit (GeneCopoeia, Inc., Rockville, MD, USA) and SYBR mix (Takara). U6 or glyceraldehyde-3-phosphate dehydrogenase (GAPDH) was used as an internal reference gene. The relative expression levels of TUG1, miR-141-3p, and ROR2 were calculated by the 2^-*ΔΔ*Ct^ method. The sequences of primers for miR-141-3p and U6 were designed and obtained from Sangon Biotech (Shanghai, China), and sequences of primers for TUG1, miR-141-3p, ROR2, U6, and GAPDH used in qRT-PCR reactions were listed: TUG1 forward (5′-GCUUGGCUUCUAUUCUGAAUCCUUU-3′), reverse (5′-AAAGGAUUCAGAAUAGAAGCCAAGC-3′); miR-141-3p forward (5′-AAGACGTACTCAGGCCATGTCC-3′), reverse (5′-GACCCAAATGTCGCAGTCAG-3′); ROR2 forward (5′-CTTGATGGCATTGTCGCTAA-3′), reverse (5′-TCCAGTGGCTGTGCTAGATG-3′); U6 forward (5′-GCTTCGGCAGCACATATACTAAAAT-3′), reverse (5′-CGCTTCACGAATTTGCGTGTCAT-3′); and GAPDH forward (5′-GACTCATGACCACAGTCCATGC-3′), reverse (5′-AGAGGCAGGGATGATGTTCTG-3′).

### 2.4. 3-(4,5-Dimethyl-2-thiazolyl)-2,5-diphenyl-2-H-tetrazolium Bromide (MTT)

An MTT reagent (Invitrogen) was added to each 96-well plate, and cells (5 × 10^3^/well) were maintained for 24 h, 48 h, and 72 h and incubated for another 4 h. After that, cell supernatant was discarded, and 200 *μ*L of DMSO (Solarbio, Beijing, China) was added to dissolve intracellular formazan crystals in each well [19]. Cell proliferation was determined at 490 nm using a microplate reader (Thermo Fisher Scientific).

### 2.5. Western Blot

RIPA buffer (Solarbio) was used to isolate total proteins from cells, and then, proteins were quantified by a NanoDrop 3000 (Thermo Fisher Scientific). Sodium dodecyl sulfate-polyacrylamide gel electrophoresis (SDS-PAGE) was used to separate proteins, and then, proteins were transferred onto polyvinylidene fluoride (PVDF) membranes. After that, membranes were blocked in skimmed milk for 2 h at 37°C followed by incubation with primary antibodies at 4°C overnight. Following 2 h incubation with secondary antibody: Goat Anti-Rabbit IgG H&L (HRP) (1 : 1000; ab205718, Abcam, Cambridge, UK), the chemiluminescence was performed by using an ECL detection kit (Beyotime, Shanghai, China). The primary antibodies were as follows: anti-ROR2 (1 : 1000; ab245456, Abcam), anti-CyclinD1 (1 : 1000; ab226977, Abcam), anti-Ki-67 (1 : 1000; ab92742, Abcam), anti-*β*-catenin (1 : 1000; ab2365, Abcam), anti-Vimentin (1 : 1000; ab137321, Abcam), and anti-GAPDH (1 : 5000; ab37168, Abcam).

### 2.6. Transwell Assay

The rate of cell migration was investigated by a transwell chamber (Corning Life Sciences, Corning, NY, USA) without a matrigel matrix, while invasion experiment was conducted with transwell chamber precoated with matrigel matrix (Corning). The lower chamber was added with RPMI-1640 medium with 10% FBS, while the transfected ox-LDL-stimulated HA-VSMCs were injected into the upper one with 100 *μ*L of serum-free medium, and the whole steps were carried out according to the manufacturer's instructions. In the end, paraformaldehyde (PFA; Sigma, St. Louis, MO, USA) was used to attach cells located on the lower surface of the upper chamber. Cells were analyzed under a microscope before staining with crystal violet.

### 2.7. Dual-Luciferase Assay

The putative binding sites of miR-141-3p and TUG1 or ROR2 were predicted by starBase software online. The amplified wild-type and the mutant fragment of TUG1 and ROR2 3′UTR were inserted into a pMIR-REPORT luciferase vector (OBio Biology, Shanghai, China) to construct luciferase reporters, namely, WT-TUG1, MUT-TUG1, WT-ROR2, and MUT-ROR2. The cotransfection of luciferase reporter and miR-141-3p or miR-NC was performed as prescribed [19]. The luciferase activity was tested using a Dual-Lucy Assay Kit (Promega, Madison, WI, USA).

### 2.8. Statistical Analysis

All data were expressed as the mean ± standard deviation (SD) and analyzed by the SPSS 17.0 software. Comparisons among different groups were analyzed using paired Student's *t*-test and one-way analysis of variance (ANOVA). A *P* value less than 0.05 was regarded as statistically significant.

## 3. Results

### 3.1. The Expression of TUG1 and miR-141-3p in Tissues of Patients with AS and in ox-LDL-Treated HA-VSMCs

**Figure 1:**
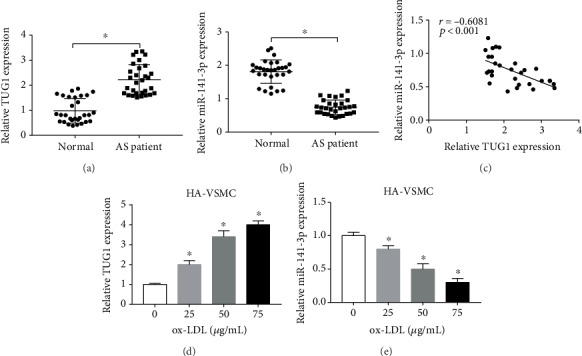
The expression of TUG1 and miR-141-3p in tissues of patients with AS and in ox-LDL-treated HA-VSMCs. (a) qRT-PCR was used to detect the expression of lncRNA TUG1 in tissues from 30 patients with atherosclerosis, compared with those from 30 healthy volunteers. (b) The expression of miR-141-3p in tissues was detected by qRT-PCR assay. (c) A correlation analysis between the expression TUG1 and miR-141-3p was shown. (d, e) HA-VSMCs were incubated with ox-LDL at various concentrations (0 *μ*g/mL, 25 *μ*g/mL, 50 *μ*g/mL, and 75 *μ*g/mL) for 24 h, followed by qRT-PCR assay of TUG1 (d) and miR-141-3p (e) expression. ^∗^*P* < 0.05.

To begin with, we examined the TUG1 level in the tissues of AS patients (*n* = 30) and healthy population (*n* = 30). The expression of TUG1 in AS tissues and normal counterparts was shown in Figure 1(a); a visible promotion in TUG1 expression was viewed in tissues of AS patients. Meanwhile, we also explored the miR-141-3p level in AS tissues. Interestingly, a reversed tendency could be observed in AS tissues, compared with that of TUG1 (Figure 1(b)). Moreover, our data suggested that there was a negative correlation between TUG1 miR-141-3p in AS tissues (Figure 1(c)). Subsequently, we used an increased dose of ox-LDL to induce HA-VSMCs for AS model construction *in vitro* and the 50 *μ*g/mL of ox-LDL for further experiments [11]. As shown in Figures 1(d) and 1(e), increased ox-LDL concentration was associated with the expression of TUG1 and miR-141-3p. To be specific, the level of TUG1 performed enhancement (Figure 1(d)) whereas decreased expression of miR-141-3p (Figure 1(e)) as the ox-LDL concentration enlarged.

### 3.2. Knockdown of TUG1 Suppressed Proliferation, Migration, Invasion, and the Expression of Metastasis-Associated Proteins in ox-LDL-Stimulated HA-VSMCs *In Vitro*

**Figure 2:**
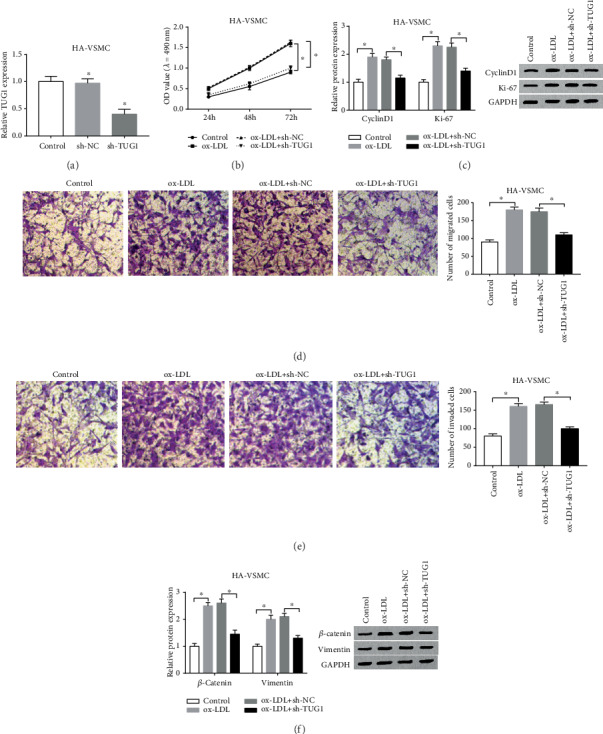
Knockdown of TUG1 suppressed proliferation, migration, invasion, and the expression of metastasis-associated proteins in ox-LDL-stimulated HA-VSMCs *in vitro*. The HA-VSMCs were transfected with sh-NC or sh-TUG1. (a) Identification in knockdown efficiency of TUG1 was analyzed by qRT-PCR. (b) The cell viability at determined times (24 h, 48 h, and 72 h) was analyzed by MTT assay in ox-LDL-stimulated HA-VSMCs. (c) The levels of proliferation-related proteins Ki-67 and CyclinD1 were confirmed by western blot. (d, e) The cell migration and invasion were evaluated by transwell assay. (f) The western blot assay was used to detect the expression of metastasis-associated proteins, *β*-catenin, and Vimentin ^∗^*P* < 0.05.

In the ox-LDL-stimulated HA-VSMCs, high cell viability, accelerated migratory and invasive abilities, and upregulated metastasis-related protein levels are thought to be imitated with early research [11]. To further investigate the biofunctional effects of TUG1 on ox-LDL-treated HA-VSMCs, we knocked down the TUG1 expression using synthesized shRNA. Afterwards, the expression level of TUG1 was downregulated after cell transfection with sh-TUG1 (Figure 2(a)). As loss-functional experiments are conducted, the cell activity was significantly reduced by determination using a MTT assay (Figure 2(b)). Then, western blot was used to detect the expression of proliferation-related proteins (CyclinD1 and Ki-67), and the results show that downregulation of TUG1 significantly decreased levels of these proteins (Figure 2(c)). By using a transwell assay, the abilities of migration and invasion were obviously limited after TUG1 knockdown (Figures 2(d) and 2(e)). Besides, the western blot results exhibited the downregulation of the expression of *β*-catenin and Vimentin in ox-LDL+sh-TUG1-treated cells compared with the cells treated with ox-LDL+sh-NC (Figure 2(f)). These results indicated that TUG1 knockdown decreased the abilities of proliferation, migration, and invasion, as well as suppressed the expression of metastasis-associated proteins, *β*-catenin, and Vimentin in ox-LDL-treated HA-VSMCs.

### 3.3. TUG1 Was a Direct Target of miR-141-3p

**Figure 3:**
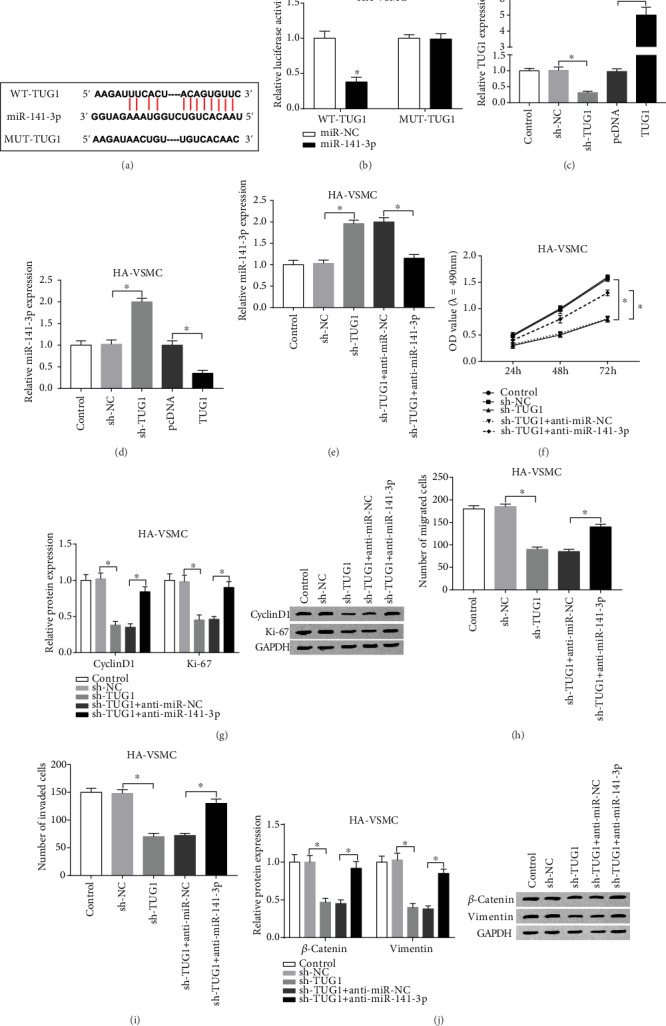
TUG1 was a direct target of miR-141-3p. (a) The putative binding sites between miR-141-3p and TUG1 were predicted by starBase. (b) The predicted sites were identified by dual-luciferase reporter assay. The ox-LDL-stimulated HA-VSMCs were transfected with pcDNA-TUG1 or negative control. (c, d) qRT-PCR was used to detect the level of TUG1 (c) or miR-141-3p (d) in each group. (f–j) The ox-LDL-administrated HA-VSMCs were transfected with sh-TUG1+anti-miR-141-3p or sh-TUG1+anti-miR-NC for further experiments. (f) MTT assay was conducted to evaluate the cell viability. (g) The proliferation-associated protein levels of Ki-67 and CyclinD1 were confirmed by western blot. (h, i) The cell migratory and invasive abilities were evaluated by transwell assay. (j) The expression of metastasis-associated proteins, *β*-catenin, and Vimentin was detected by western blot assay. ^∗^*P* < 0.05.

Next, we predicted the relationship between TUG1 and miR-141-3p by starBase, and the result showed that miR-141-3p contained complementary sequences with TUG1 (Figure 3(a)). Then, dual-luciferase reporter vectors (TUG1-WT or TUG1-MUT) were constructed with cotransfected miR-141-3p or miR-NC into ox-LDL-treated HA-VSMCs. Dual-luciferase reporter assays showed that miR-141-3p reduced the luciferase activity of TUG1-WT reporter vector, but not TUG1-MUT reporter vector (Figure 3(b)). As the loss- and gain-functional experiment confirmed by qRT-PCR, the cells witnessed an improved expression of TUG1 after the TUG1 overexpression treatment or a limited expression of TUG1 after TUG1 knockdown administration (Figure 3(c)). Furthermore, in ox-LDL-treated HA-VSMCs, the expression level of miR-141-3p was significantly upregulated by TUG1 knockdown, while the level of miR-141-3p was considerably decreased due to the overexpression of TUG (Figure 3(d)). Besides, we also evaluated the expression pattern of miR-141-3p after the cell silenced disposition upon both TUG1 and miR-141-3p; the data represented that the level of miR-141-3p was decreased in the group, namely, sh-TUG1+anti-miR-141-3p, compared with its corresponding controls (Figure 3(e)). In addition, we observed the biofunctional changes of sh-TUG1 and anti-miR-141-3p transfected into ox-LDL-treated HA-VSMCs. And the data exhibited that knockdown of miR-141-3p inverted results from silencing of TUG1 upon the cell ability proliferation (Figure 3(f)), migration (Figure 3(h)), and invasion (Figure 3(i)) and the protein expression levels (CyclinD1, Ki-67 (Figure 3(g)), *β*-catenin, and Vimentin (Figure 3(j))). Meanwhile, the low regulation of miR-141-3p also reversed the limited effects of TUG1 downregulation. Taken together, we determined that miR-141-3p was the target miRNA of TUG1. Besides, the deletion of miR-141-3p restored results from TUG1 downregulation.

### 3.4. ROR2 Was a Target Gene of miR-141-3p

**Figure 4:**
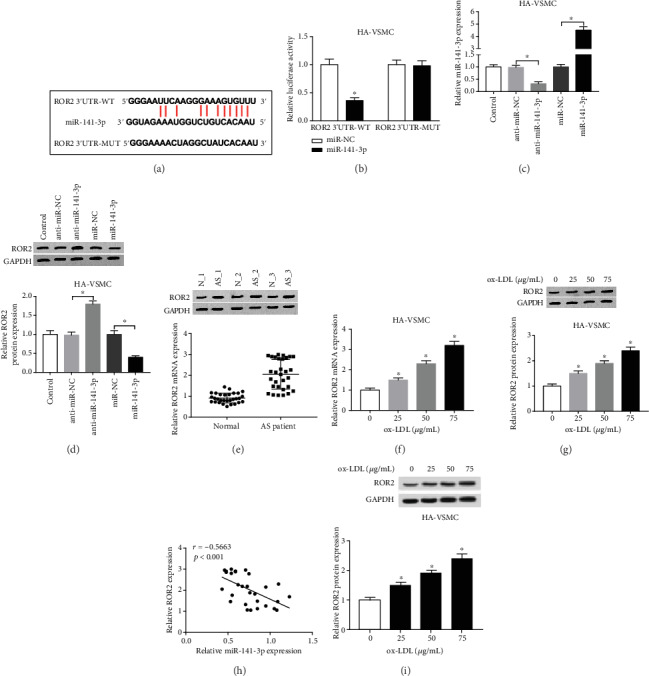
ROR2 was a target gene of miR-141-3p. (a) ROR2 was predicted by starBase as a potential target for miR-141-3p. (b) Luciferase reporter assay was conducted to verify the interaction between miR-141-3p and ROR2. (c) The expression of miR-141-3p after the loss-and-gain experiment was measured in ox-LDL-administrated HA-VSMCs using qRT-PCR. (d) The expression of ROR2 after transfection with anti-miR-141-3p or miR-141-3p was detected using western blot. ^∗^*P* < 0.05. (e, f) The relative expression of ROR2 was analyzed by qRT-PCR (e) or western blot (f) in tissues of AS patients and healthy participants. (g) The interaction between the expression of ROR2 and miR-141-3p was assayed by qRT-PCR. (h, i) The expression of ROR2 at mRNA (h) and protein (i) in HA-VSMCs treated with ox-LDL increased dose (0 *μ*g/mL, 25 *μ*g/mL, 50 *μ*g/mL, and 75 *μ*g/mL) for 24 h was tested. ^∗^*P* < 0.05.

The starBase prediction showed that ROR2 was a potential target of miR-141-3p (Figure 4(a)). Then, cotransfecting miR-141-3p or miR-NC with ROR2-WT or ROR2-MUT into ox-LDL-treated HA-VSMCs was performed. The luciferase reporter assay showed that overexpression of miR-141-3p reduced the luciferase activity with ROR2-WT, but not with ROR2-MUT (Figure 4(b)). The loss-or-gain experiment was designed by transfection with shRNA or overexpressed plasmid targeting miR-141-3p individually (Figure 4(c)). The expression of miR-141-3p was suppressed nearly twofolds in the anti-miR-141-3p group, compared with that in the anti-miR-NC group, while miR-141-3p expression in the miR-141-3p group presented over fourfold in as high as that in the miR-NC group. Besides, anti-miR-141-3p treatment significantly increased ROR2 expression, while overexpression of miR-141-3p might significantly decrease ROR2 expression (Figure 4(d)). Furthermore, we also confirmed the expression patterns in AS tissues and the expressed level of ROR2 in cells treated with different doses of ox-LDL exposure (Figures 4(e) and 4(f)); the data showed that the ROR2 expression was upregulated in AS tissues at the mRNA (Figure 4(e)) and protein (Figure 4(f)) levels and negatively correlated with that of miR-141-3p in AS tissues (Figure 4(g)). To the contrary, ROR2 expression seemed enhanced, including at mRNA (Figure 4(h)) and protein (Figure 4(i)) levels in cells, and positively correlated with the increased dose of ox-LDL administration.

### 3.5. Knockdown of miR-141-3p Inverted Functional Effects of ROR2 Deletion in ox-LDL-Stimulated HA-VSMCs *In Vitro*

**Figure 5:**
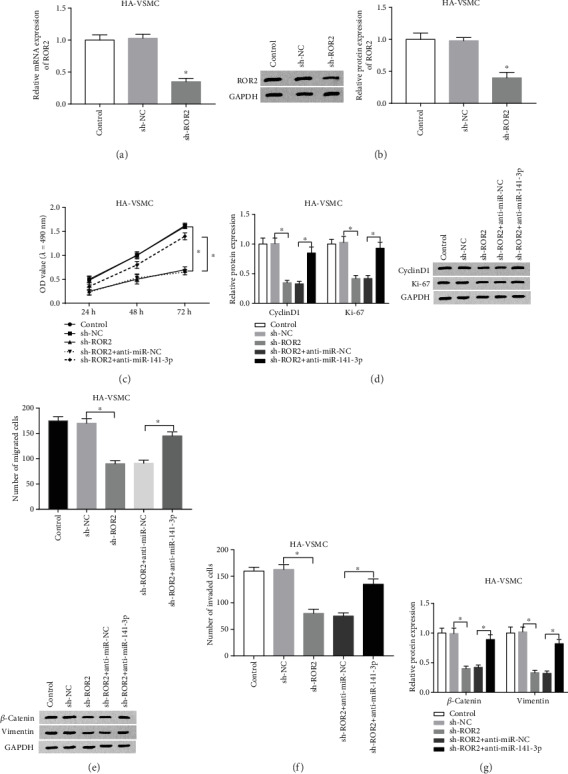
Knockdown of miR-141-3p inverted functional effects of ROR2 deletion in ox-LDL-stimulated HA-VSMCs *in vitro*. sh-NC, sh-ROR2, sh-ROR2+anti-miR-NC, and sh-ROR2+anti-miR-141-3p were transfected into ox-LDL-stimulated HA-VSMCs, separately. (a, b) The knockdown efficiency of ROR2 was confirmed by qRT-PCR (a) or western blot assay (b). (c) The cell viability was analyzed by MTT assay at stated times (24 h, 48 h, and 72 h) in ox-LDL-stimulated HA-VSMCs. (d) Western blot was used to confirm the levels of CyclinD1 and Ki-67 in cells. (e, f) Migration and invasion were evaluated by transwell assay. (g) Levels of metastasis-related proteins (*β*-catenin and Vimentin) were assessed using western blot. ^∗^*P* < 0.05.

To better explore the functional relationship between miR-141-3p and ROR2, sh-ROR2 and anti-miR-141-3p were cotransfected into ox-LDL-treated HA-VSMCs as the experimental group. Firstly, the low expression of miR-141-3p was confirmed by qRT-PCR and western blot analysis in the sh-ROR2 group (Figures 5(a) and 5(b)). Thereafter, limited cell viability in sh-ROR2+anti-miR-NC was significantly increased after deletion of miR-141-3p treatment in cells (Figure 5(c)). Then, the western blot analysis showed that the expression of CyclinD1 and Ki-67 was significantly recuperated in cells cotransfected with sh-ROR2 and anti-miR-NC (Figure 5(d)). Meanwhile, miR-141-3p silencing also promoted the abilities from ROR2 deletion on migration and invasion (Figures 5(e) and 5(f)). What is more, miR-141-3p knockdown promoted the low expression of the metastasis-associated proteins (*β*-catenin and Vimentin) from downregulation or from silencing of TUG1 in ox-LDL-treated HA-VSMCs (Figure 5(g)). These results indicated that reducing the expression of miR-141-3p inverted the biofunctional effects of sh-ROR2 production and promoted the expression of the metastasis-related proteins in ox-LDL-treated HA-VSMCs *in vitro*.

### 3.6. TUG1 Regulated ROR2 Expression by Sponging with miR-141-3p *In Vitro*

**Figure 6:**
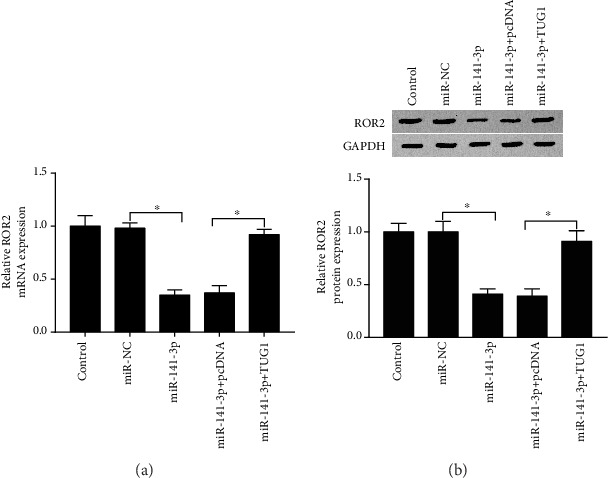
TUG1 regulated ROR2 expression by sponging with miR-141-3p *in vitro*. The expression of miR-141-3p and TUG1 was knocked down in ox-LDL-induced HA-VSMCs. (a) qRT-PCR was used to confirm the level of ROR2 in knockdown of miR-141-3p and TUG1 in ox-LDL-induced HA-VSMCs. (b) Western blot was used to confirm the level of ROR2 when silencing miR-141-3p and TUG1 in ox-LDL-induced HA-VSMCs. ^∗^*P* < 0.05.

sh-NC, sh-TUG1, sh-TUG1+anti-miR-NC, and sh-TUG1+anti-miR-141-3p were transfected into cells, individually. We examined the expression of ROR2 in ox-LDL-treated HA-VSMCs by qRT-PCR and western blot assay. Data suggested that the level of ROR2 was significantly increased after cell transfection with sh-TUG1 and anti-miR-141-3p, compared with cells transfected with sh-TUG1 and anti-miR-NC (Figures 6(a) and 6(b)). The results indicated that TUG1 mediated the ROR2 expression via regulating miR-141-3p in ox-LDL-treated HA-VSMCs.

## 4. Discussion

LncRNAs have been reported to be involved in the progression of AS [20, 21]. In the recent three years, several novel lncRNAs with abnormal expression have been defined in AS [22–24]. Yao et al. reported that lncRNA 00113 expression was significantly upregulated in the serum samples of AS, compared with healthy controls [25]. Zhao et al. found that silence of lncRNA NONMMUT002434 expression could abrogate the migration and proliferation in VSMCs [26]. However, the molecular mechanisms of lncRNAs in AS progression have not been fully elucidated.

TUG1 has been initially observed in murine retinal cells developing [27]. Subsequently, TUG1 was pervasively studied in multiple cancers, such as osteosarcoma [28], bladder cancer [29], non-small-cell lung carcinoma [30], colorectal cancer [31], and esophageal squamous cell carcinoma [32]. Several studies showed that TUG1 is involved in the process of adverse responses to cardiac disease [33]. Zhang et al. reported that ectopic expression of TUG1 contributed to cell growth, triggered inflammatory factor expression, and restrained apoptosis in ox-LDL-administrated RAW264.7 and MOVAS cells [11]. A foregone study documented that upregulation of TUG1 erased the reversed effect of tanshinol on ox-LDL-induced endothelial cell apoptosis [34]. Furthermore, several pieces of evidence have demonstrated that TUG1 overexpression remarkably promoted proliferation, migration, and cell cycle of HUVECs as well as upregulated the protein expression of *β*-catenin and c-Myc [13]. Additionally, a previous study suggested that miR-141-3p belongs to the miR-200 family, which is composed of five miRNAs and divided into two clusters on one chromosome [35]. Interestingly, a previous study uncovered that ox-LDL could suppress the expression of miR-141, and the downregulation of miR-141 boosted the proliferation of VSMCs [15].

Our data also showed the high expression of TUG1 in AS tissues and ox-LDL-treated HA-VSMCs, which was consistent with the reported paper [12]. The MTT assay showed that the viability of cells was significantly inhibited by TUG1 knockdown. The transwell assay demonstrated that sh-TUG1 inhibited the migratory and invasive abilities compared with the negative control *in vitro*. Meanwhile, identical results also could be seen in endothelial cells [13]. Also, the reducing expression of TUG1 limited the proliferation or metastasis-associated protein levels. The dual-luciferase reporter assay also confirmed miR-141-3p as a potential target of TUG1. Meanwhile, function assays revealed that miR-141-3p knockdown reversed the regulatory effects from TUG1 silencing on cells. These results above showed that TUG1 could play an essential role in proliferation, migration, invasion, and metastasis in AS progression *in vitro* through targeting miR-141-3p.

ROR2, a member of the tyrosine kinase receptor family, acts as a receptor for Wnt5a [36]. The Wnt5a/ROR2 signaling pathway primarily activates the noncanonical Wnt pathway independent of *β*-catenin, hence regulating cell proliferation and movement [37, 38]. A study by Cui et al. reported that the promotion of lncRNA 430945 in AS accelerated the biofunction of proliferation and migration in VSMCs by activating the ROR2/rhoa signaling pathway.

In our research, we identified that ROR2 was upregulated in AS tissues and ox-LDL-treated HA-VSMCs; the similar results upon ROR2 in AS were documented in the previous study [39]. Interestingly, it was also a potential target of miR-141-3p confirmed by the dual-luciferase reporter assay. Western blot showed that decreased expression of ROR2 could be seen in ox-LDL-treated HA-VSMCs transfected with miR-141-3p, whereas opposite results could be observed after the miR-141-3p knockdown. The biological function regulated by ROR2 silencing could be inverted by miR-141-3p downregulation. Meanwhile, the low expression of biomarker proteins upon proliferation and metastasis could be restored. What is more, the ROR2 protein expression was upregulated in ox-LDL-treated HA-VSMCs treated with silencing of both TUG1 and miR-141-3p in comparison with knockdown of TUG1 solely *in vitro*. That is to say, TUG1 could function as a sponge of miR-141-3p to increase ROR2 expression in ox-LDL-treated HA-VSMCs.

There were some limitations in this study; to begin with, the interaction between miR-141-3p and TUG1 or ROR2 was initially detected by the dual-luciferase reporter assay, and it should be confirmed by RNA immunoprecipitation or RNA pull-down. Besides, the results and conclusions obtained using commercial cell lines could not fully represent the actual situation *in vivo*. Thus, the AS model will be carried out for further animal experiment.

## 5. Conclusion

In conclusion, our study uncovered that TUG1, an oncogene, facilitated cell proliferation, migration, invasion, and metastasis by targeting miR-141-3p in AS progression *in vitro*. Additionally, this paper also revealed a novel axis of miR-141-3p/ROR2, supplying a novel therapeutic method for AS treatment.

## Data Availability

The data used to support the findings of this study are available from the corresponding author upon request.

## Additional Points

*Highlights*. (1) TUG1 is elevated in AS tissues and ox-LDL-treated HA-VSMCs. (2) TUG1 silencing suppresses AS procession *in vitro*. (3) TUG1 regulates ROR2 expression by sponging miR-141-3p. (4) TUG1 accelerates AS development via miR-141-3p/ROR2 axis *in vitro*.
